# A consensus statement on dual purpose pathogen surveillance systems: The always on approach

**DOI:** 10.1371/journal.pgph.0003762

**Published:** 2024-11-14

**Authors:** Helene-Mari van der Westhuizen, Srinidhi Soundararajan, Tamsin Berry, David Agus, Sergio Carmona, Philip Ma, Jessica Davis, Sarah Walker, Jolynne Mokaya, Stephen D. Bentley, Nick R. Thomson, John Silitoe, Andrew Singer, Ines Hassan, Romina Mariano, Megan Akodu, Gabriel Seidman, Nabihah Sachedina, Jonathan Edgeworth, Reshania Naidoo, Tariro Makadzange, Vladimir Choi, Renuka Gadde, Samuel V. Scarpino, Corinna Bull, Kumeren Govender, Belinda Ngongo, Hinda Ruton, Paul Pronyk, Kate Smolina, Henry Li, Dylan Barry, Sven Schaffer, Vanessa Moeder, George Gao, Derrick Crook, John Bell

**Affiliations:** 1 Global Health Security Consortium, United Kingdom; 2 Nuffield Department of Primary Care Health Sciences, University of Oxford, United Kingdom; 3 Tony Blair Institute for Global Change, United Kingdom; 4 Ellison Institute of Technology, United Kingdom; 5 Population Health Partners, United Kingdom; 6 Ellison Institute of Technology, United States of America; 7 FIND, Switzerland; 8 Prognomiq, United States of America; 9 Northeastern University, United States of America; 10 Nuffield Department of Medicine, University of Oxford, United Kingdom; 11 Wellcome Sanger Institute, United Kingdom; 12 UK Centre for Ecology and Hydrology, United Kingdom; 13 Oxford Nanopore Technologies, United Kingdom; 14 Guy’s & St Thomas’ NHS Foundation Trust, United Kingdom; 15 Ernst & Young, United Kingdom; 16 Charles River Medical Group, Zimbabwe; 17 Global Diagnostics, Clinton Health Access Initiative, United States of America; 18 Santa Fe Institute, United States of America; 19 Illumina Inc., United Kingdom; 20 Africa Quantitative Sciences, Rwanda; 21 Duke-NUS Centre for Outbreak Preparedness, Singapore, Singapore; 22 SingHealth Duke-NUS Global Health Institute, Singapore, Singapore; 23 British Columbia Centre for Disease Control; and University of British Columbia Canada; 24 The Global Commission for Post-Pandemic Policy, United Kingdom; 25 Illumina Inc., Munich, Germany; 26 Independent Global Health Consultant, United States of America; 27 D. H. Chen School of Universal Health, Zhejiang University; Hangzhou, Zhejiang Province, China; None, UNITED STATES OF AMERICA

## Introduction

The COVID-19 pandemic progressed pathogen surveillance, from improved wastewater surveillance expertise and infrastructure, increased genomic sequencing capacity, to better integration between large datasets that informed policy decisions. Yet current systems are inadequate for a future facing frequent pandemics threats [1]. There is inequitable access to pathogen surveillance globally, with existing infrastructure favouring high-income countries resulting in blind spots for collective health resilience [2]. We are concerned that political attention and investment in pandemic preparedness is waning, with missed opportunities to respond to the concurrent crises posed by antimicrobial resistance.

## Articulating the always on approach

We propose Always On - an approach that incorporates pathogen surveillance for pandemic threats with infrastructure that supports routine clinical care and public health. See Table 1 for a summary of the Always On approach. This uses surveillance data as the starting point for detecting new disease outbreaks (through tracking pathogens and identifying new variants) and monitor for emerging and re-emerging diseases but then also including a second utility to clinical care. These clinical applications include identifying drug-resistant pathogens, seasonal outbreaks of viral illnesses that may influence decisions on the use of antibiotics, for difficult-to-identify infections, and to inform public health priorities [3]. Pathogen discovery (essential for pandemic preparedness and the development of novel diagnostics, therapeutics and vaccines) can be bridged with universal or targeted pathogen detection (which could support clinical care) through sharing systems resources. This dual approach aligns with the WHO’s global genomic surveillance strategy, concepts such as collaborative surveillance and existing approaches to pathogen genomics for resource-limited settings [1, 4, 5].

**Table 1:** Key messages for always on approach.

• With a future characterised by increased pandemic threats, there is an unmet need for robust global pathogen surveillance systems. Simultaneously, there is a need for clinical diagnostics that can detect drug-resistant pathogens as part of routine care. • We propose the Always On approach, that looks for synergies between systems aimed at detecting and responding to health emergencies and the provision of routine clinical care and public health services, which could offer economic and system benefits. • While many countries are scaling back pathogen surveillance systems developed during the COVID-19 pandemic, there is an opportunity to sustain this as key health security infrastructure and build towards integrating this with tools that bring sequencing of pathogens closer to the point of patient care.

There is an economic argument to Always On. The costs of infectious diseases to society are considerable. For example the most affected continent is Africa, where infectious diseases lead to 227 million years of healthy life lost per annum, and an annual productivity loss of over $800 billion [6]. By expanding the application of pathogen surveillance technologies, it could become easier to justify financial investments, stimulating innovation and efficiencies in cost. There is opportunity to focus infrastructure developed during COVID-19 on priority endemic pathogens. Both target-specific molecular techniques and unbiased clinical metagenomics offer applications to clinical diagnosis and routine public health. Cost considerations are important, and will need to be reduced for wider use, particularly in low-resource contexts where decisions need to be made about when to deploy rapid antigen tests, multiplex PCR panels and whole genome sequencing [7]. However, a multi-pathogen focus could offer shared costs between disease programmes and promote supply chain sustainability. The Always On approach can also inform the development and supply of medicines, procurement of vaccines, and use of diagnostics based on the diseases of public health priority.

## Five guiding principles

**Fig 1:**
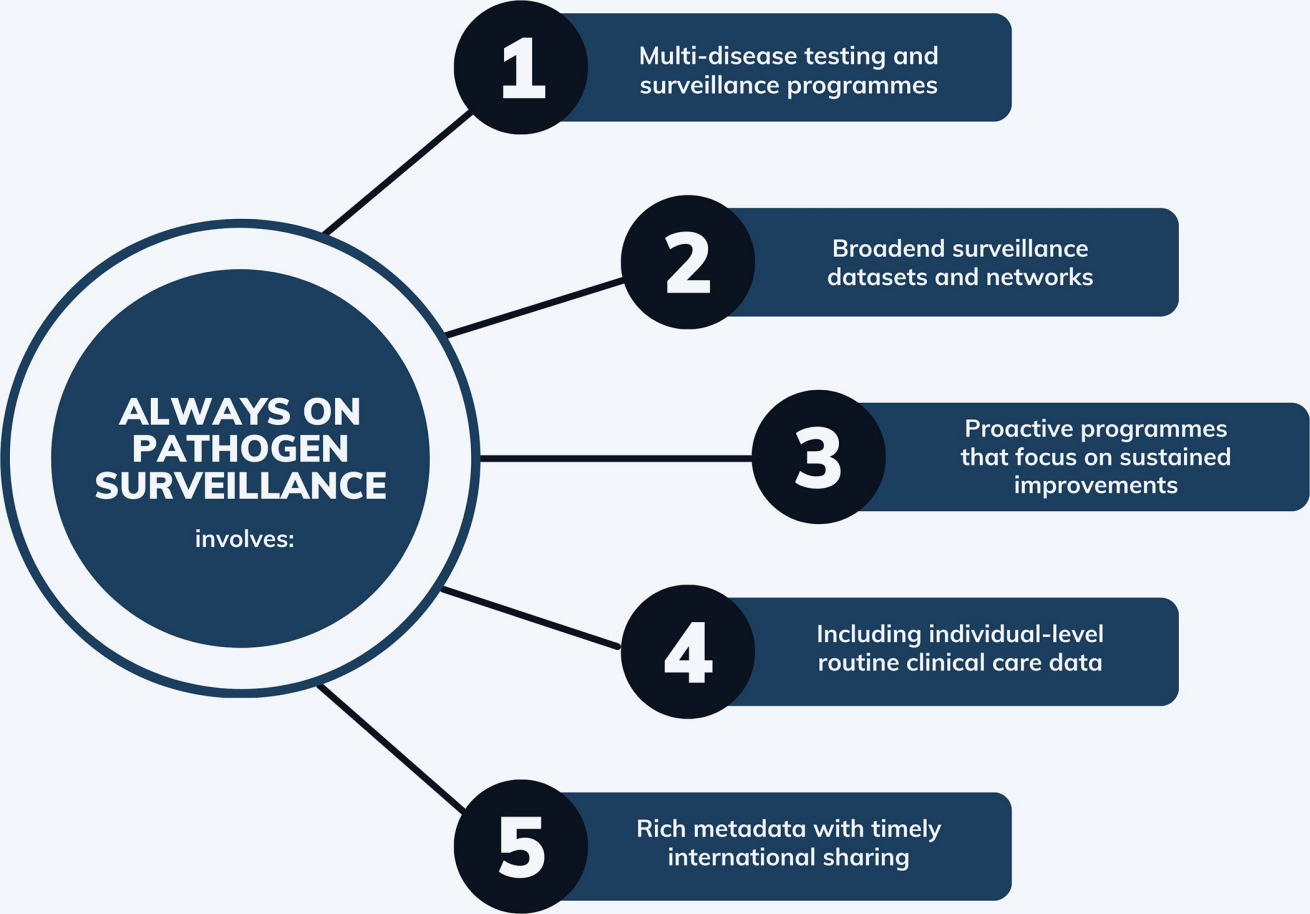
Guiding principles for an always on pathogen surveillance approach.

The Always On approach has five guiding principles, summarised in Fig 1.

### (1) Multi-disease testing and surveillance programmes

We suggest funding, by preference, surveillance infrastructure that is joined-up across diseases. This will support the development of interoperable, standardised data infrastructure and innovations in genomic testing that incorporates multiple pathogens or the ability to detect pathogens agnostically with a focus on infectious syndromes (e.g. respiratory disease). Larger procurement volumes across programmes could strengthen the supply chain infrastructure of consumables.

### (2) Broadened surveillance datasets and networks

We recommend stronger collaborations between public health practitioners and academics to broaden datasets and networks and link up existing work. Pathogen surveillance repositories (drawing on learning from clinical trial repositories that track research taking place) can co-ordinate ongoing work. Training and supporting a workforce that is not reliant on intermittent cycles of donor or project-based funding, but that are sustained in the interpandemic period is essential, enabling organisations to further develop capacity and expertise. We suggest broadening pathogen surveillance databases by capturing point-of-care or self-test results and integrating this with existing community-based outbreak investigations. Strengthened national, regional and global surveillance networks offer insights from larger datasets. This is similar value to joined-up weather radars: a larger database offers more insights when you can see what’s coming your way, not just what is upon you, already.

### (3) Proactive programmes that focus on sustained improvements

Climate change will likely worsen the global impact of infectious diseases, contributing to more frequent pandemic threats [8]. This requires a proactive approach to pathogen surveillance that is not reactive, and time-limited, but instead continuously maintained and Always On. A starting point would be for national and regional genomics strategies to articulate key disease priorities and capacity-building requirements. This should include collaboration with One Health partners, sentinel human populations and other locations of public health interest. Innovations in wastewater surveillance technology that use automated sampling to reduce the cost of collecting a sample and multiplexing of targets would be key [9]. It is important for data to be available as close to real-time as feasible, and to be actionable – either with a clinical or public health focus.

### (4) Including individual-level routine clinical care data

The clinical application of metagenomic sequencing technology holds potential for additional use of genomic infrastructure [3]. As a key enabler, there needs to be clear value to patient care (for example, predicting drug resistance phenotypes quickly and accurately, identifying difficult-to-detect infections or monitoring treatment response) in addition to what it offers the population-level outbreak response [10]. Innovations in sequencing technology, including lower cost of genomic tests and reagents, robust, closer to point-of-care tests and rapid time to result would support this and should be prioritised in future work [3]. In order to incorporate clinical care data into broader surveillance datasets, investments in the health system’s capacity to collect, manage and govern these data are needed, as well as exploring linkages with electronic health records. Should data need to cross jurisdictions, provisions should be made to ensure privacy and data governance requirements are met.

### (5) Rich metadata with co-ordinated international-level sharing, while respecting sovereignty

Data generated through routine and randomized surveillance would ideally be aggregated in national databases that integrate data across human and zoonotic pathogens of public health priority and support regular risk assessments. The data should be analysed near real-time, with best practices established to optimize signal to noise ratio, to enable decision-makers to act promptly when there is sufficient evidence to do so. Agreements on data capturing methodology (linked to phenotypic, clinical and epidemiological characterization) that support aggregations across systems are important, as well as secure data storage facilities. Data management principles, such as the FAIR approach and alignment on data sharing principles that respect data sovereignty needs to be reached [11, 12]. Large datasets could require machine learning to support data management and analysis, for which regulation (especially for electronic health record integration) has not been developed sufficiently.

In Table 2 we present a summary of high priority pathogen groupings, where genomic pathogen surveillance can have public health and clinic utility [5].

**Table 2:** High priority pathogens for genomic sequencing.

1. *Drug-resistant bacteria*: genomic sequencing can support the identification of mechanisms of drug-resistance, transmission dynamics, decisions on antimicrobial stewardship, identification of nosocomial transmission and can also inform the development and implementation of new vaccines, therapeutics and diagnostics. This also has specific application to drug-resistant *Mycobacterium tuberculosis*: in settings where there is universal access to WHO-recommended diagnostics in place, genomic sequencing plays a key role in informing targeted treatment regimens for drug-resistant tuberculosis, supporting surveillance and monitoring for the emergence of new strains [13].
2. *Respiratory viruses*: this can support outbreak detection, variant analysis (that could in turn support vaccine, therapeutics and diagnostics development and roll-out) and the detection of unknown pathogens in patients with severe respiratory illness who test negative using conventional diagnostic methods (as future pandemic threats are likely to be transmitted through the respiratory route [14]).
3. *Zoonotic spill-over pathogens*: using a One Health approach, this can detect pathogens earlier, pinpoint the animal source and dynamics of transmission, and inform outbreak control measures. This can also detect pathogen evolution earlier and inform development of new vaccines, therapeutics and diagnostics.

## Conclusion

Always On is a call for a shift in mindset strengthened by collaborations between public health practitioners, researchers, policymakers, funders, and industry to set up a more resilient pathogen surveillance system. We should aim for multi-disease approaches that better integrate with public health and routine clinical care. Global health security is too important to allow important investments to degrade or get decommissioned between crises, only to re-instate them when needed urgently again. This not only undermines health resilience but is wasteful and risky. As signatories to this statement, we are committed to support this Always On agenda.
